# From randomised trial to national implementation: employment outcomes from individual placement and support in english alcohol and drug treatment services

**DOI:** 10.3389/ijph.2026.1609141

**Published:** 2026-07-24

**Authors:** Claire Shaw, Nishanthini Oppilamany, Paul Anders, Jez Stannard, Emma Knight, Amy Baxter, John Marsden

**Affiliations:** 1 Office for Health Improvement and Disparities, Department of Health and Social Care (DSHC), London, United Kingdom; 2 Addictions Department, School of Academic Psychiatry, Institute of Psychiatry, Psychology and Neuroscience, King’s College London, London, United Kingdom

**Keywords:** alcohol and drug dependence, employment support, individual placement and support, service improvement, substance use disorder

## Abstract

**Objectives:**

To compare employment outcomes reported by a completed randomised controlled trial (IPS-AD: Individual Placement and Support - Alcohol and Drug Dependence) with those observed during the English national expansion of IPS services, and to identify factors associated with improved outcomes.

**Methods:**

This was a secondary comparative analysis of five employment outcomes – employment rate, time to employment attainment, job tenure (longest single appointment duration), total time employed and job sustainment defined as ≥13 consecutive weeks employment, in two cohorts: the IPS-AD study intervention arm (n = 843) and IPS expansion participants (n = 3,182).

**Results:**

Compared with the IPS-AD cohort, the expansion cohort showed a higher employment rate (52% versus 30%; p-value <0.001); faster employment attainment (143 versus 180 days; p-value <0.001); longer job tenure (254 versus 110 days; p-value <0.001); greater total time employed (300 versus 133 days; p-value <0.001); and higher job sustainment (76% versus 42%; p-value <0.001).

**Conclusion:**

Expansion outcomes were consistently more favourable than those observed in the IPS-AD study. Factors that may have contributed to improved outcomes included implementation and delivery enhancements, broadened eligibility criteria, and favourable environmental and economic conditions.

## Introduction

Alcohol and drug dependence is associated with profound health, social, and economic costs [1, 2]. In addition to high levels of morbidity and mortality, many individuals face long-term labour market exclusion, with unemployment and economic inactivity contributing to poverty, social marginalisation, and reduced prospects for sustained recovery [3, 4].

Employment, by contrast, plays a pivotal role in improving mental health, promoting social integration, and supporting long-term abstinence [5, 6]. Employment and recovery are mutually reinforcing [7]. Employment at treatment entry is associated with higher rates of successful treatment completion, and completing treatment is associated with improved chances of securing employment [8, 9]. Yet despite the recognised importance of employment, few labour market programmes have been consistently effective for people with alcohol or drug dependence [10, 11].

Individual Placement and Support (IPS) is an evidence-based model of supported employment developed in the United States for people with severe mental illness [12, 13]. IPS is characterised by intensive and individualised support, rapid job search and placement, integration with health and social care teams, and in-work support for employees and employers [14, 15]. The model has since been widely adopted and adapted across diverse populations, with a consistently positive evidence base [16].

In 2016, an independent review for the UK government identified IPS as a potentially effective intervention for people in treatment for alcohol or drug dependence [8]. Between 2018 and 2021, the Individual Placement and Support - Alcohol and Drug Dependence (IPS-AD) randomised controlled trial (RCT) was conducted across seven community treatment centres in England [17]. Although an earlier small-scale pilot study had tested IPS among patients receiving medication treatment for opioid use disorder [18], IPS-AD provided the first phase 3 superiority RCT evidence globally comparing IPS with treatment-as-usual (TAU) employment support for adults with alcohol or drug dependence. IPS was new to the community treatment centres that participated in IPS-AD, with all services beginning from a standing start. The study found that 30% of participants randomised to IPS achieved employment (versus 25% in the TAU arm) (adjusted odds ratio 1.29; 95% confidence interval 1.02–1.64; p-value = 0.036) [19]. The benefits of IPS were evident among those with alcohol, cannabis and some stimulant use disorders, although not among those with opioid use disorder, suggesting potential differences in the effectiveness of the model across clinical subgroups.

The IPS-AD study provided the first high-quality evidence of IPS effectiveness for people with substance dependence globally. In response, the UK government expanded IPS across England, embedding it within community alcohol and drug treatment services. Eligibility criteria were broadened: the minimum age was reduced to 16, the requirement for at least 6 months of unemployment or economic inactivity was removed, and exclusion criteria relating to more complex needs or circumstances were lifted [17].

The Department of Health and Social Care (DHSC) subsequently led a phased national expansion of IPS in yearly cohorts [20]. The expansion began in October 2019 with six IPS-AD study sites. In 2021/22, it extended to areas participating via a government initiative to combat drug use in specific areas [21], involving 11 IPS teams across 12 local authorities, and through expressions of interest, adding 14 teams across 29 local authorities. Expansion then followed government assessments of need and impact, prioritising higher-need areas: 20 teams (21 local authorities) joined in 2022/23, 12 teams (22 local authorities) in 2023/24, and 36 teams (57 local authorities) in 2024/25. By 2025, an estimated 95% of the England treatment population could access IPS. Outcome monitoring of delivery reported a 51% rate of employment attainment, with three-quarters of individuals sustaining their employment for at least 13 weeks [20].

All IPS-AD study sites underwent two fidelity reviews during the trial. At the first, all were rated ‘fair’; at the second, five improved to ‘good’ while two remained ‘fair’ [19]. Of the 50 expansion sites included in this analysis, nine completed a fidelity review during the analysis period, all achieving ‘fair’ or ‘good’. Coverage was more limited during expansion due to resource and budget constraints; however, where full reviews were not possible, most teams undertook guided self-assessments (GSA). GSAs are structured IPS Grow processes where teams assess service quality against selected fidelity items, with results reviewed by an expert to identify improvements.

In the translational context, there are important questions about factors that facilitate and hinder IPS delivery. The IPS-AD process evaluation [22] identified challenges including recruiting and retaining skilled staff; reluctance among some treatment staff to refer clients; integrating IPS so employment is seen as part of recovery; and engaging employers and providing in-work support due to perceived stigma.

Other studies have highlighted negative attitudes of referring practitioners [23]; stigma associated with mental health issues [24]; the time required to achieve high-fidelity services [12]; and a lack of mutual collaboration between IPS and treatment services [25].

Alongside these challenges, facilitators of IPS delivery have been reported. These include treatment reducing symptoms and increasing functioning for the client [24]; good quality collaboration between and integration of treatment and employment services as part of the recovery process [24–26]; IPS model implementation technical assistance and training [26, 27]; effective disclosure management of diagnoses and treatment status [28]; and secure ongoing and stable funding with supportive contracting from the funding body [26].

Wider enabling factors have also been described. Funding security, strong leadership across IPS and treatment services, support from and integration between IPS and treatment services, high fidelity with ongoing quality monitoring, and an appropriately qualified and experienced workforce have been identified as factors that contribute to the sustainability of IPS programmes beyond 2 years of service [29]. Together, these conditions create the foundation for sustainable IPS services that can deliver benefits beyond the initial years of implementation.

This study aimed to compare IPS-AD study employment outcomes with those observed during the expansion of IPS services in community alcohol and drug treatment in England. Specifically, we examined [1] employment rates [2], vocational outcomes including time to first job, job tenure, cumulative time employed, and job sustainment, and [3] subgroup patterns by sex, age, primary drug, treatment status, treatment duration, and IPS duration. By contrasting the IPS-AD study and IPS expansion cohorts, we sought to assess how lessons from the IPS-AD study translated into large-scale implementation and describe contextual factors that may have contributed to improved outcomes during national expansion.

## Methods

### Study design

We conducted a secondary comparative analysis of employment outcomes in two cohorts: (1) participants enrolled in the intervention arm of the IPS-AD study [17, 18] and (2) individuals who received IPS as part of the national expansion of the programme in England.

IPS delivered during both the IPS-AD study and the expansion programme was time limited. Participants were offered up to 9 months of employment support, with an additional period of up to 4 months of in-work support for those who obtained employment, resulting in a total support duration of between nine and 13 months, depending on employment attainment and individual preference.

### Participants

Eligible participants were adults engaged in community-based treatment for alcohol or drug dependence in England who were seeking competitive employment. Two cohorts were included: 843 individuals recruited to the IPS-AD study intervention arm between May 2018 and September 2019, and 3,182 individuals who received IPS during the national expansion between October 2019 and May 2023. Participants provided informed written consent for a set of their personal identifiers (National Insurance number, postcode, date of birth, and initials) to be shared with HM Revenue and Customs (HMRC) for data linkage.

### Data linkage

For both datasets, participants’ identifiers were matched to HMRC employment and tax contribution records, with initials used for verification. HMRC matched 83% of the IPS-AD study cohort and 98% of the expansion cohort. Data were enriched through the Pay As You Earn (PAYE) income tax system and self-assessment returns to capture both salaried and self-employed participants. HMRC returned anonymised linked datasets providing employment outcome data for 18 months before and 18 months after IPS enrolment. IPS-AD study data linkage took place in April 2021 and included the seven study sites. Data linkage for the IPS expansion cohort was completed in January 2025 and included 50 IPS teams operating in 66 English local authorities (43% of all local authorities). Although the expansion rollout was almost complete in January 2025, the 18-month follow-up period and accounting for data lag meant that the expansion linkage included only IPS teams with individuals who had enrolled in IPS before the end of May 2023.

### Creation of the combined dataset

To enable direct comparison of outcomes, variables common to both datasets were identified and harmonised. Where definitions differed, values were recoded to achieve consistency. For example, the IPS expansion dataset drug group variable contained six categories, which were collapsed into three categories to align with the IPS-AD study. After harmonisation, the two datasets were merged to create a single analytic sample. Variables that could not be aligned across datasets were excluded from the combined file.

### Outcomes

For both cohorts, the primary outcome was attainment of competitive employment during an 18-month follow-up period, defined as being employed for at least 1 day in the open labour market. Follow-up began at randomisation for the IPS-AD study cohort and at admission for the IPS expansion cohort. Secondary vocational outcomes were: (1) time from IPS enrolment to first job; (2) job tenure, defined as the duration of the longest single period of employment; (3) cumulative days employed across all jobs in the 18-month follow-up period; and (4) job sustainment, defined as employment in a single job for at least 13 consecutive weeks.

### Statistical analysis

Descriptive statistics summarised participant characteristics for each cohort. Differences between groups were assessed using the chi-square test for categorical variables. For the primary outcome, attainment of employment was compared between the IPS-AD study and IPS expansion cohorts using the chi-square test for categorical variables. Secondary outcomes were analysed using Chi-square tests and Mann–Whitney U tests, as appropriate. The threshold for statistical significance was set at p-value <0.05. All analyses were conducted using IBM SPSS, version 29.0 (IBM Corp, Armonk, NY, USA).

## Results

### Participant characteristics

Table 1 summarises the characteristics of the IPS-AD study and IPS expansion participants at study enrolment and IPS expansion admission, respectively. The two cohorts did not differ by sex (p-value = 0.335). The IPS expansion cohort included a greater proportion of those aged under 25, those with alcohol dependence, those who successfully completed their treatment, those in treatment for over 12 months, and those engaged in IPS for under 2 months (all p-values <0.001).

**TABLE 1:** Characteristics of participants (England, UK, IPS-AD study 2018–2019, IPS expansion 2019–2023).

Variables	IPS-AD study (%)	IPS expansion (%)	p-value^a^
Sex^†^			
Males	588 (69.8)	2,276 (71.5)	0.335
Females	254 (30.1)	906 (28.5)	​
Age			
Under 25	36 (4.3)	263 (8.3)	<0.001
25 years +	807 (95.7)	2,919 (91.7)	​
Primary drug			
Opioid	421 (49.9)	1,063 (33.4)	<0.001
Other drug	118 (14.0)	484 (15.2)	​
Alcohol	304 (36.1)	1,635 (51.4)	​
Treatment status^†^			
Still in treatment	389 (46.1)	1,032 (32.4)	<0.001
Successful completion	224 (26.6)	1,442 (45.3)	​
Unsuccessful exit	146 (17.3)	707 (22.2)	​
Treatment length^†^			
Up to 3 months	217 (25.7)	397 (12.5)	<0.001
Up to 6 months	116 (13.8)	549 (17.3)	​
Up to 12 months	157 (18.6)	710 (22.3)	​
More than 12 months	332 (39.4)	1,526 (48.0)	​
IPS length^†^			
Up to 2 months	81 (9.6)	556 (17.5)	<0.001
More than 2 months	761 (90.4)	2,626 (82.5)	​

^a^ p-value from Pearson Chi-squared test.

Data are n (%).

^†^ Measure with missing data.

Sex: 1 participant in IPS-AD, study; Treatment status: 84 participants in IPS-AD, study and 1 participant in IPS, expansion; Treatment length: 21 participants in IPS-AD, study; Time in IPS: 1 participant in IPS-AD, study.

It was not possible to directly compare the previous work history of the two cohorts. IPS-AD study inclusion criteria required participants to have been unemployed or economically inactive for at least 6 months prior to enrolment, based on self-report. Our analysis shows that 8% of the IPS expansion cohort were employed in the 6 months before admission. Given that the criterion for IPS-AD study inclusion was self-report, it is possible that prior employment history was inaccurately reported by some participants [30].

### Successful employment

Compared with the IPS-AD study cohort, the IPS expansion cohort was more likely to gain competitive employment (52% versus 30%; p-value <0.001) (Table 2). This difference was consistent across subgroup analyses. Within both cohorts, employment attainment was more common among females, those with alcohol dependence, those who successfully completed treatment, and those engaged in IPS for more than 2 months (all p-values <0.001).

**TABLE 2:** Rates of successful employment (England, UK, IPS-AD study 2018–2019, IPS expansion 2019–2023).

Variables	IPS-AD study (%)	IPS expansion (%)	p-value^a^
All	207 (29.7)	1,641 (51.6)	<0.001
Sex			
Males	139 (28.6)	1,145 (50.3)	<0.001
Females	68 (32.2)	496 (54.7)	<0.001
Age			
Under 25	7 (25.9)	169 (64.3)	<0.001
25 years +	200 (29.9)	1,472 (50.4)	<0.001
Primary drug			
Opioid	80 (22.9)	438 (41.2)	<0.001
Other drug	31 (33.7)	268 (55.4)	<0.001
Alcohol	96 (37.5)	935 (57.2)	<0.001
Treatment status			
Still in treatment	76 (23.0)	462 (44.8)	<0.001
Successful completion	74 (38.5)	865 (60.0)	<0.001
Unsuccessful exit	31 (26.5)	314 (44.4)	<0.001
Treatment length			
Up to 3 months	60 (34.5)	226 (56.9)	<0.001
Up to 6 months	40 (41.2)	320 (58.3)	0.002
Up to 12 months	36 (27.1)	415 (58.5)	<0.001
More than 12 months	65 (23.3)	680 (44.6)	<0.001
IPS length			
Up to 2 months	8 (11.8)	230 (41.4)	<0.001
More than 2 months	199 (31.6)	1,411 (53.7)	<0.001

^a^ p-value from Pearson Chi-squared test.

Data are n (%).

Compared with the IPS-AD study cohort, the proportion of participants successfully gaining employment was at least 50% higher across all primary drug groups in the IPS expansion cohort. The largest increase was among those with opioid dependence (80%), followed by other drug dependence (64%) and alcohol dependence (53%).

**FIGURE 1:**
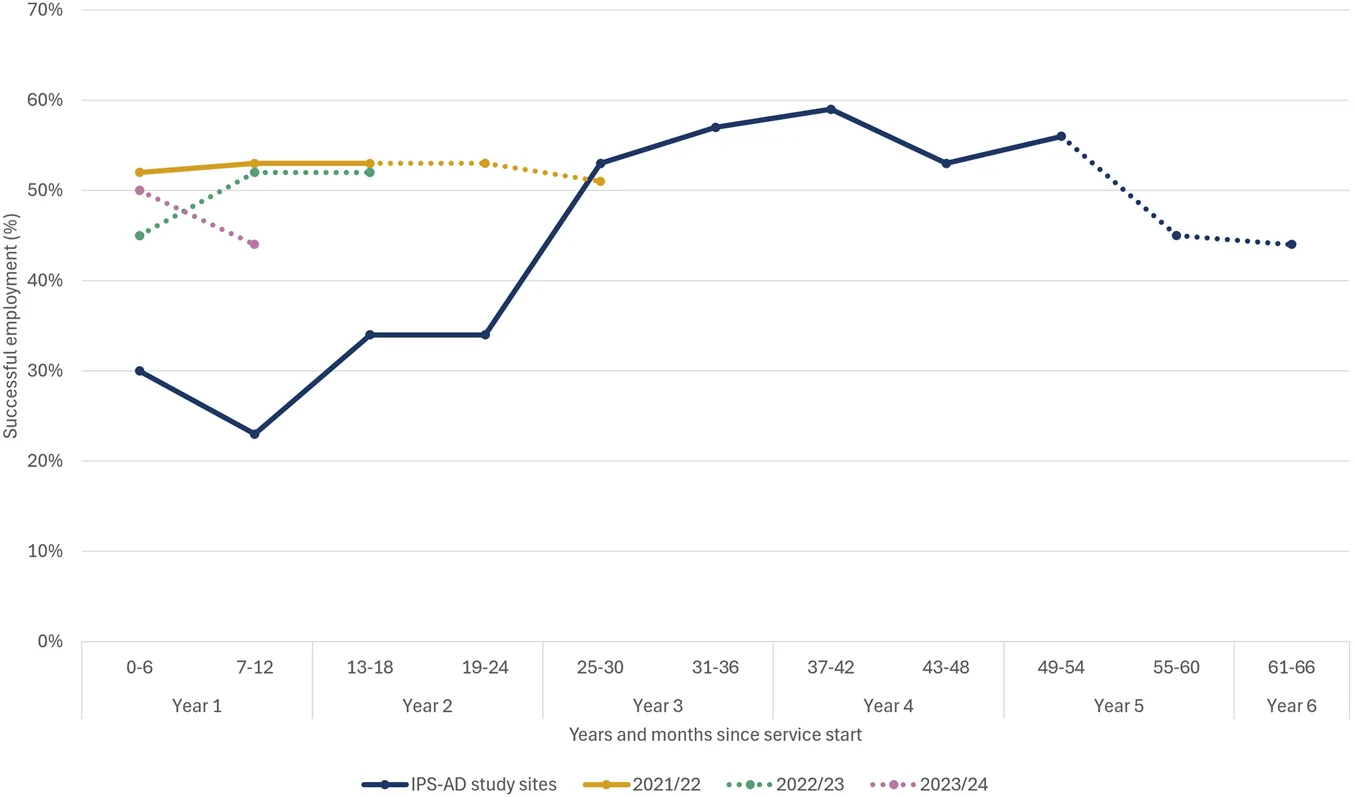
Rate of successful employment by wave of Individual Placement and Support implementation (England, UK, IPS-AD study 2018–2019, IPS expansion 2019–2023). Footnotes to accompany Figure 1. Note: Dotted lines denote time periods where participants have not yet completed their 18-month follow-up and rates of successful completion will increase during these periods in future data linkage rounds. For the IPS-AD study sites time periods 0–6 to 13–18 represent the IPS-AD study period. Data are %.

Figure 1 presents successful employment rates by wave of IPS implementation in 6-month intervals. IPS-AD study site employment rates were lower during the study and in the 6 months immediately following it, with marked improvement from 24 months onwards. In contrast, IPS expansion sites showed higher rates of successful employment from the early stages of implementation.

### Vocational outcomes

The IPS expansion cohort entered employment more quickly than the IPS-AD study cohort (143 versus 180 days; p-value <0.001), a difference replicated across most subgroup analyses with the exception of those aged under 25 (p-value = 0.383), those with other drug dependence (p-value = 0.302), those in treatment up to 3 months (p-value = 0.273) and up to 12 months (p-value = 0.084) and those in IPS for up to 2 months (p-value = 0.861) (Table 3).

**TABLE 3:** Vocational outcomes of participants who successfully obtained employment (England, UK, IPS-AD study 2018–2019, IPS expansion 2019–2023).

​	Time to first job (in days) Mean ± SD			Job tenure (in days) Mean ± SD			Total time employed (in days) Mean ± SD			Jobs sustained n (%)		
​	IPS-AD study	IPS expansion	p-value^a^	IPS-AD study	IPS expansion	p-value^a^	IPS-AD study	IPS expansion	p-value^a^	IPS-AD study	IPS expansion	p-value^b^
All	179.68 ± 142.60	143.38 ± 127.44	<0.001	110.17 ± 120.91	254.27 ± 171.68	<0.001	132.58 ± 141.64	300.36 ± 182.07	<0.001	87 (42.0)	1,240 (75.6)	<0.001
Sex												
Male	175.01 ± 139.10	140.86 ± 124.74	0.003	117.40 ±124.63	244.76 ± 172.07	<0.001	138.84 ± 143.93	292.05 ± 182.89	<0.001	63 (45.3)	845 (73.8)	<0.001
Female	189.21 ± 150.10	149.23 ± 133.44	0.022	95.40 ± 112.35	275.83 ± 168.99	<0.001	119.78 ± 136.98	319.17 ± 178.97	<0.001	24 (35.3)	395 (79.6)	<0.001
Age												
Under 25	103.57 ± 131.19	139.47 ± 129.32	0.383	103.00 ± 63.40	220.69 ± 159.62	0.062	135.57 ± 74.39	271.73 ± 176.94	0.048	4 (57.1)	124 (73.4)	0.345
25 Years+	182.34 ± 142.54	143.87 ± 127.25	<0.001	110.43 ± 122.51	258.22 ± 172.67	<0.001	132.48 ± 143.53	303.72 ± 182.43	<0.001	83 (41.5)	1,116 (75.8)	<0.001
Primary drug												
Opioid	183.70 ± 144.89	144.13 ± 127.99	0.019	106.30 ± 126.45	249.65 ± 170.98	<0.001	116.69 ± 134.85	290.85 ± 179.66	<0.001	32 (40.0)	328 (74.9)	<0.001
Other drug	181.03 ± 156.91	152.27 ± 140.69	0.302	83.61 ± 80.47	214.48 ± 165.64	<0.001	115.03 ± 116.45	263.07 ± 182.65	<0.001	10 (32.3)	188 (70.1)	<0.001
Alcohol	175.89 ± 137.22	140.40 ± 123.03	0.006	121.98 ± 126.39	267.83 ± 172.00	<0.001	151.49 ± 153.04	315.46 ± 181.37	<0.001	45 (46.9)	724 (77.4)	<0.001
Treatment status												
Still in treatment	179.22 ± 140.70	145.30 ± 135.44	0.017	100.41 ± 127.70	256.11 ± 174.56	<0.001	110.03 ± 134.74	296.39 ± 184.50	<0.001	28 (36.8)	344 (74.5)	<0.001
Successful completion	182.55 ± 147.43	143.68 ± 125.13	0.030	108.57 ± 112.64	268.61 ± 167.95	<0.001	133.39 ± 138.38	318.39 ± 175.70	<0.001	31 (41.9)	696 (80.5)	<0.001
Unsuccessful exit	162.10 ± 145.25	139.91 ± 122.78	0.500	117.35 ± 98.60	211.70 ± 171.33	0.006	151.71 ± 123.31	255.95 ± 188.45	0.005	16 (51.6)	200 (63.7)	0.185
Treatment length												
Up to 3 months	165.67 ± 143.49	138.94 ± 123.63	0.273	100.78 ± 96.98	251.58 ± 180.07	<0.001	131.95 ± 122.54	296.11 ± 185.50	<0.001	26 (43.3)	169 (74.8)	<0.001
Up to 6 months	195.47 ± 147.36	137.83 ± 128.22	0.008	122.03 ± 145.99	247.30 ± 168.20	<0.001	142.60 ± 169.20	300.92 ± 181.75	<0.001	15 (37.5)	243 (75.9)	<0.001
Up to 12 months	175.86 ± 134.62	145.66 ± 129.29	0.084	124.75 ± 125.87	248.17 ± 164.59	<0.001	154.31 ± 151.30	297.41 ± 177.55	<0.001	17 (47.2)	316 (76.1)	<0.001
More than 12 months	182.52 ± 143.28	143.26 ± 127.33	0.043	99.29 ± 122.17	262.33 ± 174.74	<0.001	105.26 ± 124.71	303.36 ± 184.17	<0.001	25 (38.5)	512 (75.3)	<0.001
IPS length												
Up to 2 months	127.75 ± 106.74	131.12 ± 135.77	0.861	99.00 ± 88.32	220.65 ± 174.15	0.045	116.88 ± 105.37	268.50 ± 187.31	0.023	4 (50.0)	154 (67.0)	0.318
More than 2 months	181.76 ± 143.66	145.28 ± 126.06	<0.001	110.62 ± 122.18	259.69 ± 170.72	<0.001	133.21 ± 143.07	305.49 ± 180.76	<0.001	83 (41.7)	1,086 (77.0)	<0.001

^a^ p-value from Mann-Whitney U test.

^b^ p-value from Pearson Chi-squared test.

Job tenure was longer in the IPS expansion cohort (254 versus 110 days; p-value <0.001), with consistent findings across all subgroups except those aged under 25 years (p-value = 0.062).

Total time in employment was greater for the IPS expansion cohort (300 versus 133 days; p-value <0.001), with differences observed across all subgroup analyses. Job sustainment was achieved more often in the IPS expansion cohort than the IPS-AD cohort (76% versus 42%; p-value <0.001). This finding was consistent across most subgroups in the expansion cohort, except for those aged under 25 years (p-value = 0.345), those who left treatment unsuccessfully (p-value = 0.185) and those engaged in IPS for under 2 months (p-value = 0.318).

## Discussion

This study compared the outcomes of the IPS-AD study with those from the subsequent national expansion of IPS in England. The expansion cohort was more likely to gain employment (and from the outset), did so more quickly, and had more sustained employment and total days of employment than the IPS-AD study cohort.

Successful employment rates were higher across all expansion subgroups than in the IPS-AD cohort, with increases across all primary drug groups. The largest improvement was in the opioid dependence group, a key policy finding, given the size and clinical importance of this group, and the IPS-AD study’s earlier conclusion that enhanced IPS may be more suitable for this group [19].

Across secondary vocational outcomes, expansion subgroups generally outperformed the IPS-AD cohort, though not consistently. Those under 25 did not secure employment faster, have longer single periods of employment, or achieve higher job sustainment. Similarly, individuals who exited treatment unsuccessfully or engaged in IPS for less than 2 months did not gain employment faster or achieve higher job sustainment rates.

While the IPS-AD study found no evidence that IPS was associated with successful completion of drug treatment [19], this analysis showed expansion participants who completed treatment had higher employment rates, secured jobs faster and sustained employment longer than IPS-AD counterparts. This aligns with evidence on the mutually reinforcing relationship between employment and recovery [8, 9].

Figure 1 does not show the full heterogeneity in IPS-AD study site performance during the trial, with outcomes ranging from large positive to negligible or negative effects [19]. By contrast, the expansion period shows clear convergence across sites, indicating a levelling up of service delivery. Teams achieved broadly similar outcomes, suggesting that with sufficient time, support, and sustained implementation, previously underperforming services can reach the levels of higher-performing teams. This is reflected in the more uniform performance of IPS-AD sites during the expansion period.

This study was not designed to examine empirically the implementation mechanisms underlying differences between the IPS-AD study and the expansion. It therefore cannot identify which factors, if any, were causally responsible for the improved expansion outcomes, and selection or other biases may exist. However, findings from an independent IPS expansion evaluation [31] and DHSC programme experience suggest that improved implementation and delivery, broader eligibility criteria, and more favourable environmental and economic conditions may have contributed, though this cannot be confirmed.

International studies show that secure funding, supportive contracting, IPS model implementation technical assistance and training, leadership in the IPS field, and ongoing service quality monitoring facilitate engagement and sustainment of IPS programmes [23, 25–27, 29]. We suggest that DHSC, as funder and lead organisation, developed a stronger understanding of delivery through the IPS-AD study and its evaluation, and applied these lessons, alongside stronger national programme management support, during the expansion [31].

During IPS-AD, DHSC supported delivery through monthly one-to-one calls with team leaders, all-site conference calls, and email summaries of management information and data quality. While COVID-19 and associated restrictions posed significant challenges for the delivery of drug and alcohol treatment, IPS delivery, and employer engagement, they also accelerated the adoption of online meeting technologies. This support model was strengthened during expansion, with improvements informed by DHSC learning from IPS-AD and insights from external evaluation [31].

Ongoing support and service quality monitoring now include weekly national calls open to all IPS staff and IPS Grow representatives, four-weekly check-ins, monthly performance and data quality reports, and a shared performance management system for DHSC, IPS leaders, IPS Grow, and commissioners. This system provides near real-time feedback and encourages ‘friendly competition’. Online meetings have also enabled a strong community of practice among geographically dispersed teams, often operating within competing treatment provider organisations.

One notable expansion development was DHSC’s commissioning of the UK not-for-profit Social Finance to extend IPS Grow, the national technical support programme for evidence-based IPS across the health system. IPS Grow provided implementation support, workforce training, a performance management system, and independent fidelity reviews. This support, unavailable at the start of IPS-AD, may have strengthened expansion delivery through added team support and fidelity assurance. From early in the expansion, IPS Grow worked closely with DHSC to target support and fidelity assurance to teams with greatest need, reflecting evidence on the importance of fidelity for employment outcomes [32]. An appropriately qualified and experienced workforce has been identified as a factor that facilitates IPS programme sustainment [29]. In the UK, IPS operates in secondary mental health and some primary care services in addition to community alcohol and drug treatment. As the IPS expansion rolled out, providers in alcohol and drug treatment benefited from a larger pool of qualified and experienced potential recruits with experience of the IPS model.

Close integration between treatment and employment services supports IPS engagement and sustainability [24–26, 29, 31]. In England, several large treatment providers also deliver IPS across multiple areas, ensuring this integration. Some have invested central resources and created national IPS coordinator roles to provide leadership, support, monitoring, and technical assistance and training [26, 27, 29, 31].

The IPS-AD study sites had no previous experience of IPS. The expansion of IPS had two characteristics that enabled effective peer-to-peer support and cultivated a positive culture within the workforce: increased IPS provision within regions, and increased provision within provider organisations [31]. The former meant that more experienced (or more effective) IPS teams could be paired with newer teams to host site visits and provide informal, generally short-term, mentoring. The latter enabled organisations with multiple IPS teams to leverage that experience as well as economies of scale to introduce peer support opportunities entirely independent of those facilitated by DHSC and IPS Grow.

As a result of broadening the eligibility criteria and removing IPS-AD study exclusion criteria [17], the expansion cohort represented a wider spectrum of needs and circumstances and accessibility of IPS was increased, including for people who had been unemployed or economically inactive for less than 6 months and therefore had more recent employment experience.

The wider economic context during the expansion may have also facilitated employment outcomes. In the period following the COVID-19 pandemic, the ratio of job vacancies to unemployed people rose to unprecedented levels, reaching 1:0.96 in June 2022 [33]. This tight labour market likely improved opportunities for IPS performance, although it was driven in part by reduced labour supply rather than increased demand [34].

At the same time, the end of the UK’s European Union transition period altered labour market dynamics [35, 36]. IPS teams reported that employers in sectors previously reliant on EU workers - including manufacturing, distribution, retail, hospitality, and social care - reported difficulties in recruitment. IPS teams suggested that these shortages may have made employers more open to hiring candidates who might previously have been marginalised.

These findings suggest that evidence-based employment support can deliver good outcomes at scale. Individuals who engage with IPS have a strong chance of securing employment. For IPS services and policymakers, the findings show that outcomes can not only be maintained but enhanced at scale, particularly with implementation support, fidelity assurance, workforce development and performance management monitoring.

### Strengths and limitations

The study has several strengths. The use of HMRC administrative data provided robust employment outcomes ascertainment, and we achieved high match rates. We used a large national expansion cohort, encompassing 50 IPS teams across 66 local authorities, allowing outcomes to be assessed across diverse geographical, service and labour market contexts.

Our findings should be considered in the light of several limitations. The primary purpose of the present report was descriptive and comparative rather than causal, and therefore comparisons between the IPS-AD study and expansion should not be interpreted causally and observed differences may partly reflect compositional and contextual differences between populations and services. The two cohorts were not matched on baseline characteristics and were recruited in different time periods, meaning that contextual factors such as COVID-19 and labour market shifts could have influenced results. The IPS-AD study sample was smaller, limiting power for subgroup analyses. Data on the characteristics of the expansion cohort were more limited than those available for the IPS-AD cohort, owing to differences in data linkage consent procedures that constrained further sub-group analyses, including ethnicity. In addition, HMRC data do not capture employment type, hours worked, or informal work (‘cash-in-hand’ jobs), potentially underestimating total employment.

### Challenges and opportunities

Despite improved performance during IPS expansion, challenges remain and, potentially, consequent opportunities to improve outcomes further.

Secure funding is cited as a facilitator of programme sustainability [26, 29]. Although DHSC gained a better understanding of optimal funding allocation during the IPS expansion, multiple short-term funding rounds have occurred during the expansion phase, with funding allocations of a minimum of one and a maximum of 3 years, which may have adversely affected IPS staff recruitment and retention.

Compared with IPS in other English services, such as secondary mental health, IPS teams in alcohol and drug treatment cover smaller geographical areas (103 teams cover 97% of England), have smaller team sizes (typically 1 team leader and 2 IPS employment specialists) and a smaller overall workforce. Smaller IPS teams in community alcohol and drug treatment may reduce resilience and limit career progression beyond team leader, negatively affecting retention, particularly at team leader level.

The large number of IPS teams has increased costs for implementation, technical support, and fidelity assurance. As a result, most teams rely on GSA [37] rather than full IPS 25-item fidelity reviews [32, 38], and fidelity scores cannot be included in this analysis as a measure of implementation quality. Although all sites are intended to undergo at least one full review, early reviews were prioritised for areas most likely to benefit from earlier review and are therefore not representative of overall performance. Together, these challenges may widen performance gaps between teams and limit further improvement; addressing them could enhance outcomes.

The IPS-AD study was pivotal in providing the first rigorous evidence that IPS could be adapted for people with alcohol and drug dependence in England and demonstrated the potential for significant gains in employment outcomes, making the policy case for national expansion. With this rollout, large cohorts of service users are now represented in routine datasets, creating opportunities to move beyond the descriptive analysis presented here. We are currently developing further research using a Target Trial Emulation approach [39], which frames observational data as if generated through randomisation, using linked administrative data, sequential risk-set methods, and inverse probability of treatment weighting. By constructing a comparison group of eligible non-initiators, more rigorous causal estimates of employment outcomes can be derived, affording the potential for a real-world, large-scale, and robust evaluation of IPS outcomes in future studies.

### Conclusion

IPS expansion employment outcomes were consistently better than those observed in the IPS-AD study. Factors that may have contributed to improved outcomes included implementation and delivery enhancements, broadened eligibility criteria, and favourable environmental and economic conditions.
